# Common clinical laboratory features among women with polycystic ovary syndrome and metabolic syndrome

**DOI:** 10.25122/jml-2023-0057

**Published:** 2023-08

**Authors:** Volodymyr Artyomenko, Valentyna Velychko, Daria Lahoda, Halyna Danylchuk

**Affiliations:** 1 Department of Obstetrics and Gynecology, Odesa National Medical University, Odesa, Ukraine; 2 Department of Family Medicine and Polyclinic Therapy, Odesa National Medical University, Odesa, Ukraine

**Keywords:** metabolic syndrome, polycystic ovary syndrome, obesity, insulin resistance, pre-diabetes, comorbid pathology, triglycerides, lipids

## Abstract

Women with polycystic ovary syndrome (PCOS) have a high prevalence of metabolic syndrome (MS), with rates up to 33%. This is associated with long-term consequences such as cardiovascular diseases, type 2 diabetes mellitus (T2DM), cancer, sleep apnea, and psychological issues. The prevalence of MS worldwide is often associated with obesity and T2DM, yet regional variations are reported. In this study, 122 women consulting general practice and family medicine physicians were evaluated, revealing a BMI exceeding 30 kg/m^2^. Among MS criteria, the most common diagnoses were T2DM in 29 patients, insulin resistance (IR) in 36, arterial hypertension (AH) in 51, reduced HDL levels in 53, and elevated triglycerides in 39. Further analysis revealed 16 unique combinations of MS components in these patients, with 75% of PCOS cases exhibiting three MS components and 25% having four. Additionally, research indicated that most women with PCOS face persistent, treatment-resistant obesity, with a notably higher BMI (ρ=0.87; r=0.76). These findings highlight the multifactorial nature of PCOS and MS etiology.

## INTRODUCTION

Metabolic syndrome (MS) is a cluster of symptoms that includes conditions such as obesity (body mass index (BMI) of up to 30 kg/m^2^), high blood pressure (AH), disorders in carbohydrate metabolism (pre-diabetes or diabetes mellitus (DM)), dyslipidemia (elevated triglycerides (TG) or low levels of high-density lipoprotein cholesterol (HDL)). If a patient meets at least three of these criteria, they are diagnosed with MS. Typically, clinical understanding of MS focuses on two aspects: cardiovascular and endocrinological factors, with particular attention to insulin resistance and its consequences.

Approximately 10-18% of women of reproductive age are affected by polycystic ovary syndrome (PCOS) [1]. Women with PCOS have a high prevalence of metabolic syndrome, with rates reaching up to 33%. MS is linked to long-term consequences such as cardiovascular diseases, type 2 diabetes mellitus (T2DM) cancer, sleep apnea, and psychological issues [2].

The prevalence of metabolic syndrome worldwide is often linked to obesity and type 2 diabetes mellitus, but studies have shown varying results in different countries. For example, Hirode *et al*. found that in the United States, the prevalence of MS significantly increases with age, from 19.5% among people aged 20 to 39 years to 48.6% among those aged 60 and older [3]. It has also been discovered that MS raises the risk of developing T2DM by 5 times and that PCOS is a significant non-modifiable risk factor for these patients [4, 5]. Women with PCOS who initially have normal blood sugar levels have a greater chance of developing impaired glucose tolerance, with around 16% developing carbohydrate metabolism disorders yearly. Women with initial impaired glucose tolerance have a 2% risk of progressing to type 2 diabetes mellitus per year, and within 6 years, this risk can rise to 54% [6, 7]. Additionally, Pattnaik *et al*. found that PCOS increases the risk of developing gestational diabetes mellitus [8]. Women with PCOS face a twofold increased risk of developing coronary heart disease (CHD) and stroke. Additionally, women who have MS have a 3-6 times greater chance of developing CHD and a 12% increased likelihood of premature death [9-11]. The co-occurrence of MS and PCOS further exacerbates these risks.

There is a connection between polycystic ovary syndrome (PCOS) and an increased risk of developing endometrial cancer [12]. However, it is challenging to determine the independent impact of PCOS on endometrial cancer since other factors such as diabetes mellitus, hypertension, obesity, chronic anovulation, and hyperestrogenemia may also contribute to the development of this cancer. It is important to mention that there is no significant link between polycystic ovary syndrome and ovarian or breast cancer [13]. On the other hand, metabolic syndrome is connected to a higher probability of developing endometrial cancer and an increased incidence of postmenopausal breast cancer, pancreatic cancer, and colorectal cancer [14, 15]. The combined risks of PCOS and MS are the same as above.

Women diagnosed with PCOS have a higher occurrence of depression than the general population, which does not appear to be affected by their BMI [16]. Furthermore, women with PCOS are also susceptible to anxiety, eating disorders, and poor relationships [16]. Individuals with metabolic syndrome often experience depression, particularly with symptoms such as fatigue [16]. While inflammation is connected to the emergence of depression, there is still uncertainty about the precise mechanisms behind this relationship.

It is crucial for the healthcare system to implement a systematic evaluation that can detect and treat MS in women of reproductive age. For this assessment to be practical in a clinical setting, it should prioritize risk factors, standardize evaluation methods, and determine the frequency of follow-up testing.

At present, there is a need to enhance the understanding of general practitioners about the relationship between PCOS and MS in women who seek medical care. Therefore, this study aimed to explore various clinical laboratory characteristics in this population.

## MATERIAL AND METHODS

### Study design and participants

In this study, 122 women who consulted a physician of general practice and family medicine were examined, all with a BMI exceeding 30 kg/m^2^ and an average age of 32±2.31 years. Out of these, 78 were diagnosed with metabolic syndrome (MS), and 24 had both MS and polycystic ovary syndrome (PCOS). The study included women aged 18 to 40 who had a body mass index (BMI) greater than 30 kg/m^2^ and had experienced a regular menstrual cycle for at least the previous two years, with voluntarily signed informed consent. The exclusion criteria were refusal to participate in the study, absence of a regular menstrual cycle for the past 2 years, pregnancy, current intake of specific medications such as antihypertensive, hypolipidemic drugs, or diabetes medications, and those with hypertension, hyperlipidemia, and hyperglycemia based on medication usage. Additionally, women taking Metformin, those with thyroid dysfunction, mental disorders, or chronic diseases and exacerbations were also excluded from the study.

### Assessments and measurements

All participants underwent a comprehensive screening for PCOS and MS. Standard measurements included BMI, waist circumference, and blood pressure. Blood samples were drawn between 08:00 and 10:00 to assess fasting glucose, insulin, glycated hemoglobin, HDL cholesterol, and triglyceride (TG) levels [17].

Female patients were evaluated for MS prevalence based on NCEP/ATP III criteria and WHO criteria [18]. According to the NCEP/ATP III criteria, women were diagnosed with MS if they met at least three of the following criteria: increased waist circumference (more than 88 cm), low levels of HDL cholesterol (less than 1.3 mmol/l in women), high levels of serum triglycerides (more than 1.7 mmol/l), high blood pressure (more than 130/85 mmHg), and high fasting blood glucose (more than 110 mg/dl or 6.1 mmol/l). On the other hand, women were diagnosed with MS based on WHO criteria if they had one primary criterion, such as DM or insulin resistance, impaired glucose tolerance, plus two secondary criteria, such as obesity (BMI>30), AH, dyslipidemia (evaluated by low HDL cholesterol and TG) [19, 20].

Insulin resistance was measured using the quantitative insulin-sensitivity check index (QUICKI) [21]. Pre-diabetes or type 2 DM were diagnosed based on the criteria set forth by the American Diabetes Association (ADA) [22]. The study employed the ECLIA method on the Cobas 6000 analyzer by Roche Diagnostics (Switzerland) to measure insulin levels. Blood glucose levels were measured using the enzyme (hexokinase) method. The cholesterol esterase method was used to determine HDL cholesterol after selectively precipitating lipoproteins containing apolipoprotein B with a polyanion solution. Additionally, following lipase hydrolysis, triglycerides were enzymatically measured as glycerol using an automatic chemical analyzer by Roche Diagnostics (Switzerland).

To diagnose PCOS, the Rotterdam Conference criteria were used, and pelvic or intravaginal sonography was performed in accordance with the 2018 international evidence-based guideline for the assessment and management of polycystic ovary syndrome.

To rule out non-classical 21-hydroxylase deficiency, a normal level of 17-hydroxyprogesterone in circulation was used, and the presence of Cushing's syndrome was determined based on clinical findings and/or urinary cortisol assessment. Women with very high androgen levels or abnormalities on computed tomography or magnetic resonance imaging were excluded from the study to eliminate the possibility of androgen-secreting neoplasms. Anovulation was defined as a serum progesterone level in the luteal or second phase of the cycle <10 nmol/l. For women who reported having "normal" menstruation, at least two consecutive cycles with low blood serum progesterone were required to establish a diagnosis of anovulation. Menstrual cycles shorter than 25 days or longer than 34 days were considered abnormal.

### Blood sample collection

Ten milliliters of blood were collected from each patient. Five milliliters were used for the HbA1c assay in tubes containing EDTA. Four milliliters were collected for lipid profiles, and one milliliter was used for fasting blood sugar analysis. Glucose assay analysis was conducted immediately, while the remaining samples were centrifuged and stored at -21°C until measurement.

### Statistical analysis

Data were processed using Microsoft Excel 2020. The study utilized various statistical analysis methods, including dispersion, correlation, regression, and discriminant analysis. Both parametric and non-parametric analysis methods were used to process the statistical results. Descriptive characteristics for indicators measured on a quantitative scale were provided using median and mean values. For the comparative analysis of independent groups, the Student's t-test was used for even samples, provided that the data distribution was normal and homoscedastic. On the other hand, the Mann-Whitney test was utilized for heteroscedastic data with a different distribution type. The study examined the relationship between attributes by performing correlation analysis using the Spearman method. In addition, the Pearson chi-square test was used to evaluate the association between qualitative and quantitative attributes.

## RESULTS

In accordance with the study protocol, all participants in this research were females with a BMI equal to or exceeding 30 kg/m^2^. These patients underwent screening for MS per the criteria outlined in Table 1.

**Table 1:** Baseline characteristics and metabolic parameters of participants (n=122)

Parameter		General group (n=122)	MS (n=78)	MS+PCOS (n=24)
BMI, kg/m^2^		32,24±0,54	31,97±0,14	33,56±0,61^
Fasting glucose level, mmol/l		6,1±0,43	5,96±0,21	6,12±0,18
Insulin, mIU/ml		21,74±1,25	22,05±2,01	22,14±1,15
HbA1, %		6,25±0,21	6,18±0,16	6,67±0,31
Blood pressure, mm mm Hg	Systolic	138,37±2,56	145,52±1,42*	142,62±1,63
	Diastolic	79,46±2,41	75,25±1,73	77,91±1,69
HDL, mmol/l		0,99±0,17	1,51±0,12*	1,49±0,23**
TG, mmol/l		1,62±0,14	2,18±0,09*	2,05±0,12**

* p^GG-MS^<0,05; **p^GG-MS-PCOS^<0,05; ^p^MS-PCOS^<0,05

Table 1 summarizes the results of the study, indicating that patient values did not exceed the reference values on average. Participants had first-degree obesity based on their BMI, and those with MS+PCOS had a significantly higher BMI than those with MS. Moreover, women with MS had the highest level of systolic blood pressure.

The analysis of laboratory parameters with regard to the lipid profile showed that female patients with MS and the combination of PCOS+MS had significantly higher levels of triglycerides and lower levels of high-density lipoprotein cholesterol (l<0.05; l<0.05, respectively). However, when diagnosing metabolic syndrome (MS) in each specific case, the study found that the majority of women had MS, namely 78 versus 44 women with obesity without metabolic syndrome.

Among the diseases falling under the MS criteria, the most common diseases diagnosed (n=78) were type 2 DM in 29 female patients, insulin resistance (IR) in 36 female patients, AH in 51 female patients, dyslipidemia in the form of a reduced HDL level in 53 female patients, and an elevated TG in 39 female patients. Various combinations of these MS components were observed among female patients, with the combination of arterial hypertension, low HDL levels, and carbohydrate metabolism disorder being the most frequent.

**Figure 1:**
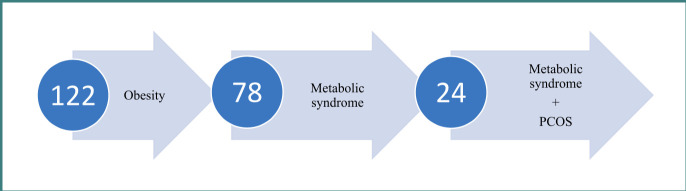
The occurrence of polycystic ovary syndrome in relation to metabolic syndrome

All patients, regardless of MS status, were referred for intravaginal sonographic examination of the pelvic organs, and the data obtained are presented in Figure 1.

Figure 1 illustrates that a significant portion of examined women with PCOS also had MS, accounting for 30.76% (n=24) of the 78 cases. Six (13.63%) female patients had a combination of PCOS and obesity without MS.

**Figure 2:**
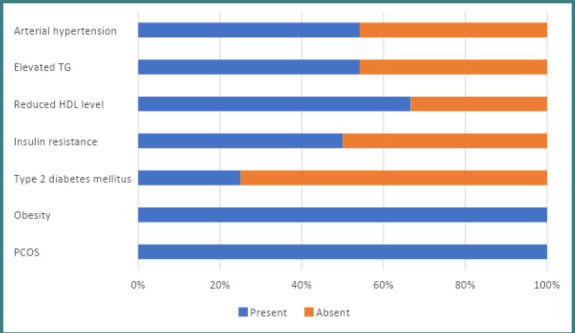
Prevalence of various metabolic syndrome components among participants

Figure 2 displays the distribution of female patients with different MS components. A comprehensive analysis revealed 16 distinct combinations of these components. An in-depth examination further indicated that 75% (n=18) of female patients with PCOS exhibited a combination of three MS components, while 25% had a combination of four MS components (n=6). Furthermore, the study revealed that most women diagnosed with PCOS had persistent obesity and a higher body mass index (BMI) over an extended period, with a strong positive correlation (ρ=0.87; r=0.76). These findings confirm the multifactorial nature of both PCOS and MS.

## DISCUSSION

Research indicates that insulin resistance (IR) and subsequent hyperinsulinemia frequently play a crucial role in the development of polycystic ovary syndrome (PCOS) [23]. Insulin controls the functioning of metabolic and mitogenic pathways that operate independently [24, 25]. This could account for the contradictory insulin sensitivity patterns observed in various tissues, including peripheral tissue resistance and preservation of sensitivity in the ovarian cortex [26]. Despite substantial evidence supporting the link between IR and PCOS, it is still unclear whether this relationship is cause-and-effect, whether it is mutually coexistent, or whether IR exacerbates PCOS symptoms [27]. Therefore, our study observed IR in 50% of female patients with PCOS+MS and 46.15% of female patients with MS.

The effect of atherogenic dyslipidemia on the course and development of PCOS in women with MS was also examined. The findings suggest that insulin resistance causes more free fatty acids to be diverted from adipose tissue to the liver. This leads to the production of very low-density lipoproteins (VLDL), increased levels of triglycerides and apolipoprotein B, and decreased levels of HDL [28]. This alteration in lipid parameters results in a form of atherogenic dyslipidemia.

The chronic, low-grade systemic inflammation is linked to polycystic ovary syndrome. In addition to genetic abnormalities, proinflammatory cytokines such as tumor necrosis factor-alpha (TNFα) and interleukin-6 (IL-6) are known to be induced by obesity and high-glycemic diets. This is characteristic of women with metabolic syndrome. Our study confirmed the link between PCOS and lipid metabolism disorders, as 70.83% of women with PCOS and metabolic syndrome had low levels of HDL, and 54.17% had high levels of TG [29].

Obesity is commonly observed in women with PCOS as a coexisting condition. This is believed to result from a combination of factors such as genetic susceptibility, unhealthy dietary habits, and a lack of physical activity, which exacerbate underlying metabolic abnormalities. Additionally, research has shown that a mother's lifestyle choices can have an impact on the health of her female offspring, who may later become mothers themselves [30, 31]. Hyperinsulinemia and hypersensitivity of the ovarian cyst to insulin cause an increase in free androgen levels. Elevated levels of androgens in the blood can make people more prone to developing central obesity. This can exacerbate existing insulin resistance and dyslipidemia [32, 33].

High blood pressure is a common health problem in people with metabolic syndrome. This is thought to be due to increased activity of the sympathetic nervous system and the renin-angiotensin-aldosterone system [34]. This affects the increase in the level of insulin and free fatty acids. The arterial hypertension development is also facilitated by concomitant dysfunction of the vascular endothelium [35].

Upon analyzing all the data related to the etiopathogenesis of PCOS, it is apparent that our understanding of the metabolic syndrome associated with PCOS has evolved. It is no longer considered simple ovarian dysfunction but a complex and multifactorial condition that affects multiple systems in the body and has greater metabolic consequences than previously thought.

## CONCLUSION

Metabolic syndrome is frequently not diagnosed despite being highly prevalent. Women with PCOS have a high prevalence of metabolic syndrome, with our study showing that 30.76% of women with PCOS met the global criteria for metabolic syndrome. It is crucial to establish screening guidelines to enable early detection and implement effective secondary prevention measures. The only universally accepted intervention is lifestyle modification.

## Data Availability

The corresponding author can provide additional data upon reasonable request.

## Personal thanks

Our team would like to thank LLC Artemis LTD (Ukraine) for their invaluable assistance in interpreting and guiding us through the manuscript.
